# Social Cognition and Mild Cognitive Impairment in Mid-Stage Parkinson’s Disease

**DOI:** 10.3390/bs14020101

**Published:** 2024-01-29

**Authors:** Roberto Fernández-Fernández, Guillermo Lahera, Beatriz Fernández-Rodríguez, Pasqualina Guida, Clara Trompeta, David Mata-Marín, Carmen Gasca-Salas

**Affiliations:** 1 HM CINAC (Centro Integral de Neurociencias Abarca Campal), Hospital Universitario HM Puerta del Sur, HM Hospitales, 28938 Madrid, Spain; 2 Hospital Universitario Infanta Cristina, Parla, 28981 Madrid, Spain; 3 PhD Program in Health Sciences, University of Alcalá de Henares, 28054 Alcalá de Henares, Spain; 4 Department of Medicine and Medical Specialities, University of Alcala, 28054 Alcalá de Henares, Spain; 5 Ramón y Cajal Institute of Sanitary Research (IRYCIS), 28034 Madrid, Spain; 6 Psychiatry Service, Center for Biomedical Research in the Mental Health Network, University Hospital Príncipe de Asturias, 28805 Alcalá de Henares, Spain; 7 PhD Program in Neuroscience, Cajal Institute, Autónoma de Madrid University, 28029 Madrid, Spain; 8 Network Center for Biomedical Research on Neurodegenerative Diseases (CIBERNED), Instituto Carlos III, 28031 Madrid, Spain; 9 School of Medicine, University CEU-San Pablo, 28003 Madrid, Spain

**Keywords:** theory of mind, social cognition, Parkinson’s disease, mild cognitive impairment

## Abstract

Mild cognitive impairment (MCI) is a relevant non-motor feature in Parkinson’s disease (PD). Social cognition (SC) is a cognitive domain that refers to the ability to decode others’ intentions and to guide behavior in social contexts. We aimed to compare SC performance in mid-stage PD patients compared to a healthy population and according to their cognitive state. Fifty-two PD patients were classified as being cognitively normal (PD-CN) or having mild cognitive impairment (PD-MCI) following the Movement Disorder Society (MDS) Level II criteria. SC assessment included facial emotion recognition (FER), affective and cognitive theory of mind (ToM), and self-monitoring (RSMS test). Twenty-seven age-matched healthy controls (HC) were enrolled. PD-MCI patients scored worse than HC on affective and cognitive ToM task scores. Only cognitive ToM scores were significantly lower when compared with the PD-MCI and PD-CN groups. We found no differences in FER or self-monitoring performance. There were significant correlations between cognitive ToM and executive functions, memory, language, and attention, whereas FER and affective ToM correlated with memory. Our findings indicates that SC is normal in cognitively unimpaired and non-depressed mid-stage PD patients, whereas a decline in affective and cognitive ToM is linked to the presence of MCI.

## 1. Introduction

Parkinson’s disease (PD) is the second most common neurodegenerative disorder after Alzheimer disease (AD). In 2017, the Global Burden of Disease (GBD) study documented 1.02 million incident cases of Parkinson’s disease [1]. The cardinal motor features are bradykinesia, tremor, and rigidity with a typical response to dopaminergic treatment [2]. In some cases, patients may develop motor fluctuations, with the clinical effect of medication diminishing over time, leading to an OFF state and a decline in motor function. Non-motor manifestations such as cognition may also fluctuate, emphasizing dopamine’s crucial role in cognitive functions, as reported in the literature [3]. However, PD also comprises a heterogeneous spectrum of non-motor symptoms that contribute greatly to overall disease burden [4]. Cognitive decline is one of the most studied non-motor aspects of PD. Prevalence studies have found that mild cognitive impairment (MCI) is present even in newly diagnosed PD patients [5,6], and dementia is common, especially in late stages. It has been found that 83% of patients are affected after 20 years of disease [7]. This makes dementia one of the most important non-motor manifestations of PD, with an important impact on the quality of life [8,9].

MCI has been recognized as an intermediate phase between normal cognition and dementia in the general population [10] as well as in PD [11,12]. Its relevance is such that the Movement Disorder Society (MDS) appointed a task force to outline the diagnostic criteria for MCI in Parkinson’s disease, which may impact the following cognitive domains: attention and working memory, executive functions, visuospatial skills, memory, and language [11]. Its cognitive correlates have been extensively examined as a risk factor for PD dementia (PDD) [13,14] and have shown a moderate but robust effect on conversion of PD-MCI to PDD [15].

Social cognition (SC) is a domain that can also be affected in PD patients but has been less frequently studied [16]. It refers to the ability to decode others’ intentions and behaviors and use those social cues to guide behavior in social contexts. This cognitive domain mainly comprises three clusters: facial emotion recognition (FER), theory of mind (ToM, also known as social understanding), and social decision making [17]. ToM refers to our ability to comprehend our own and others’ cognitive states, such as thoughts, beliefs, and intentions (referred to as “cognitive ToM”), as well as affective states like emotions or feelings (“affective ToM”), and to anticipate others’ actions based on these mental representations [18]. In addition, we are interested in other aspects of SC, such as self-monitoring, the ability to work out social and emotional cues, whose impairment can be observed in other neurodegenerative diseases such as frontotemporal dementia [19]. Self-monitoring in PD has been investigated in only one study, regardless of cognitive state [20]. SC impairment has been described in the early stages of PD in numerous studies [21,22,23]. 

Most of the studies about SC in PD have been focused on non-demented patients; therefore, they do not differentiate among cognitively normal individuals and those with MCI. Two recent studies in patients with PD-MCI showed poorer scores in SC tasks, such as FER tasks and ToM, than in PD patients with preserved cognition [24,25]. By contrast, a recent study showed FER and ToM impairment often occurred in the absence of impairment in any other cognitive domain [26].

Moreover, depression has been related to social cognitive deficits in the general population [27,28] and in PD patients [29]. In addition, higher levels of depression have been associated with MCI in PD [30,31]. However, previous studies on PD-MCI patients did not consider it as a potential confounder of SC performance.

In this study, we aimed to compare SC performance in mid-stage PD patients compared to healthy population and according to their cognitive state and to further study the relationship between SC and other cognitive domains. 

## 2. Materials and Methods

### 2.1. Subjects

We performed an observational, case–control, cross-sectional study. Fifty-two patients with PD diagnosed according to the United Kingdom Parkinson’s Disease Society Brain Bank Criteria [32] were recruited by convenience sampling at HM CINAC, Hospital Universitario HM Puerta del Sur, from May 2017 to April 2021 to undergo social, neuropsychological, and neuropsychiatric evaluation. Patients had a disease duration of less than 10 years and an age of disease onset greater than 50 years. Twenty-seven age- and gender-matched healthy controls (HC) were also enrolled for statistical comparison. Exclusion criteria for all participants were coexistence of severe cerebrovascular disease, metabolic disease, active oncologic process, severe depression (score over 20) at baseline measured by Geriatric Depression Scale (GDS-30) [33], presence of alterations in magnetic resonance imaging that indicate other causes of cognitive impairment, and any previous neurosurgical intervention. Subjects with dementia were excluded following the Movement Disorder Society criteria [11]. The study received the HM Puerta del Sur Ethics Committee approval (16.09.0942E1-GH), and all subjects provided signed informed-consent prior to investigation.

### 2.2. Clinical Assessment

Demographic and clinical data included age, gender, disease duration, educational level, and disease severity evaluated using the MDS-Unified Parkinson’s Disease Rating Scale (MDS-UPDRS) part III (motor score) and levodopa equivalent daily dose (LEDD). The LEDD condenses the antiparkinsonian medication into a single number according to a standardized method [34]. The GDS and the Starkstein apathy scale (SAS) were performed in all patients. The entire evaluation was performed during the on-medication state so that we avoid clinical fluctuations that could affect comparisons between patients. The battery of tests was performed in one session, or two in case of signs of fatigue, and the order of the tests was randomized.

### 2.3. Neuropsychological Evaluation

In addition to SC assessment, a neuropsychological battery was performed, including the following tests: attention/working memory (digit span forward and backward [35], a test that requires the patient to repeat numbers in either identical or reverse sequence as read by the evaluator; Trail Making Test A (TMTA) [36], which requires rapid linkage of fifteen sequentially numbered circles); executive function (Stroop test inhibition time (TMTB) [36], a similar test that requires alternating between connecting numbers and letters in order; and phonemic fluency [37], where the subject is asked to name as many items as he or she can recall for a specific phoneme (i.e., /p/)); language (Boston Naming Test [38], a test in which the examiner presents a series of black and white line drawings, enabling the participant to identify and name each item; semantic fluency [37], which asks the subject to name as many items as he or she can recall in a category fluency (i.e., animals)); memory (Consortium to Establish a Registry for Alzheimer’s Disease (CERAD) [39], delayed recall/recognition for verbal memory, a test that consists of three learning trials, a delayed recall trial and a recognition trial; and Wechsler Memory Scale for visual memory [40], designed to assess the capacity for recalling designs from memory and accurately reproducing and recognizing them); visuospatial function (Judgment of Line Orientation [JLO] [41], a test that measures accuracy of angular orientation through assessments of a pair of angled lines, based on their visual congruence; Visual Object and Space Perception Battery for visuospatial domain (VOSP) [42], which evaluates visuospatial function focusing on one component of visual perception, while minimizing other cognitive functions; and a copy of the Rey–Osterrieth complex figure (ROCF) [43], which evaluates visuospatial constructional ability, wherein the patient is instructed to copy a complex geometric figure onto a blank sheet of paper.). Patients were classified as cognitively normal (PD-CN) or as having mild cognitive impairment (PD-MCI) following the MDS Level II criteria [11], which implies comprehensive neuropsychological testing with two tests for each of the five cognitive domains. Impairment was required to be present on at least two tests in one or different cognitive domains [11].

### 2.4. Social Cognition Assessment

SC was evaluated during the on-medication state and included measures of FER, affective ToM, cognitive ToM, and a self-monitoring test. In order to rule out difficulties in face perception, we first used the Benton Facial Recognition Test (BFRT) as a potential prerequisite for the successful interpretation of emotional expressions. In this test, the participant was required to match a target face with either one face from the same viewpoint (6 items) or three out of six faces with varying viewpoints and lighting conditions (16 items). The maximum score on the task is 54, and a score less than or equal to 38 implies impairment of individual face-matching ability [44]. For FER assessment, we used the Karolinska Directed Emotional Faces (KDEF) test [45], which comprised 64 chosen photographs, 8 for each emotion (anger, disgust, fear, happiness, sadness, surprise), and 16 depicting neutral faces (no expression) from the electronic database [46,47]. Participants had to identify which emotion was expressed in each photograph, and each correct response scored one point, with a maximum of 64 points. Affective ToM was assessed with the revised version of the Reading the Mind in The Eyes Test (RMET) [48,49,50] that consists of 36 photographs of the eye region of male and female subjects. The participants were asked to choose which of the four options better described the intentions and emotions of the subjects in the photographs. As in the previous test, each photograph scored one point, with a maximum of 36. For exploring cognitive ToM we used the Theory of Mind Picture Stories Task (ToM stories) [51] that uses a cartoon picture story consisting of four pictures that comprise a first-order false belief, a second-order false belief, and a tactical deception. The participants had to order the four pictures in the correct chronological sequence. If they failed, a correction was made, and the story was correctly presented before ToM testing. Participants were then asked about the story with questions about the ability to infer the mental states of the characters in the story. Scores were classified into a maximum global rate of 59 points, a correct sequencing rate (maximum score of 36), and a correct questionnaire score (maximum score of 23); two points were given to the first and last cards and 1 point to the rest and for each question. Finally, the Revised Self-Monitoring Scale (RSMS) [52] consists of 13 items to be answered by the informant, covering the capacity to regulate one’s (patient) behavior in a social context. The answers are on a 6-point Likert-type scale, and the patients are more likely to adapt to a social context when the score is higher.

### 2.5. Statistical Analysis

Clinical and neuropsychological variables were compared between the three groups: HC, PD-CN, and PD-MCI. Normally distributed variables were compared by ANOVA followed by Bonferroni post hoc correction; variables with non-parametric distribution were studied by the Kruskal–Wallis test followed by Dwass–Steel–Critchlow–Fligner pairwise comparisons post hoc test. In the case of categorical variables, the Chi-square test was applied. 

Finally, Pearson correlation test or Spearman rank correlation coefficients (according to normality of distribution) and corresponding 95% confidence intervals (CIs) were performed, followed by Bonferroni correction, to assess the correlation between SC variables and neuropsychiatric and neuropsychological measures. Statistical analyses were performed with STATA software version 17 (STATA Corporation, College Station, TX, USA). 

## 3. Results

### 3.1. Demographic, Clinical, and Neuropsychiatric Assessment

As a remainder, fifty-two non-demented PD patients and twenty-seven HC were enrolled in this study. Six patients (2 PD-CN and 4 PD-MCI) were removed for FER analysis because they scored ≤38 on the BFRT. Following the MDS PD-MCI diagnosis Level II criteria, thirty-three patients were classified as PD-CN, while nineteen were classified as PD-MCI. The mean age of the participants was 67.2 years (SD = 5.04), with no significant differences between groups. PD-MCI patients only differed from HC subjects in apathy, showing higher scores. PD-MCI patients also scored worse than PD-CN patients in apathy and in the MDS-UPDRS motor score (*p* < 0.05). There were no other significant differences in demographic or neuropsychiatric variables between groups. The demographic results are shown in Table 1.

### 3.2. Social Cognition Assessment According to Classification Groups

A Kruskal–Wallis test was performed to determine if there were statistically significant differences among diagnostic groups on SC tests (Table 2). In case of cognitive ToM, we found significant differences in ToM stories total scores (*F* = 6.85, *p* < 0.05) between PD-MCI groups (M = 44.5, SD = 10.76) compared to PD-CN (M = 50.5, SD = 9.07) and HC (M = 51.73, SD = 6.12). When we analyzed the ToM stories sequencing and questionnaire scores separately, only questionnaire scores remained statistically significant (*F* = 11.14, *p* < 0.05) for the same groups. 

However, in the case of affective ToM and FER, we observed significant differences in RMET test (F = 8.43, *p* < 0.05), but only between PD-MCI (M = 18.4, SD = 3.24) and HC (M = 22.4, SD = 4.59). The analysis of the total KDEF score and analysis of each emotion individually showed no significant results (*F* = 2.23, *p* = 0.32). 

### 3.3. Correlations between Neuropsychological and Neuropsychiatric Evaluation and Social Cognition Assessment 

We calculated the correlation coefficient of the relationship between neuropsychological and neuropsychiatric variables with SC.

After Bonferroni correction, the following statistically significant correlations were found: (1) ToM task total score correlated with TMT-A (r = −0.52; *p* < 0.05) (attention), TMT-B (r = −0.59; *p* < 0.05) and phonemic fluency (r = 0.54; *p* < 0.05) (executive function), Boston Naming Test (r = 0.63; *p* < 0.05) (language), and WMS-IV (r = 0.56; *p* < 0.05) (visual memory); (2) affective ToM correlated with CERAD (r = 0.52; *p* < 0.05) (verbal memory); and (3) FER correlated with Stroop test inhibition (r = −0.50; *p* < 0.05) (executive function) and CERAD (r = 0.58; *p* < 0.05) (verbal memory) (Table 3).

## 4. Discussion

The primary purpose of this study was to examine SC abilities in non-demented mid-stage PD patients with and without cognitive impairment (PD-CN and PD-MCI). Our results revealed that PD-MCI patients show worse global (affective and cognitive) ToM performance than HC. In contrast, when we compared PD-MCI and PD-CN patients, only ToM task scores (more specifically in the questionnaire part) showed differences, with no significant results for affective ToM. We found no differences when comparing FER and RSMS performance among groups. There was a moderate negative correlation between cognitive ToM and executive functions, as well as positive with memory (WMS), language and attention (TMT-A) cognitive tests, whereas FER and affective ToM, only were moderately positively correlated with memory function.

Our results support previous data that reported both cognitive and affective ToM are impaired in PD-MCI patients [24,25]. In our study, patients with severe clinical depression were excluded, given its association with SC impairment in general population [27] and PD patients [29]. In comparison with previous studies, disease duration was relatively homogeneous in our sample (mean disease duration of 5.56 ± 2.09 years) that allows the study of SC in a well-established population of late-onset mid-stage PD patients. Finally, in our study, we have applied the MDS Level II criteria for the diagnosis of PD-MCI [11], which seem to have the best balance of sensitivity, specificity, and diagnostic accuracy, as well as a better predictive value for the development of PDD for PD-MCI patients compared to patients with normal cognition [53]. 

It has been proposed that cognitive ToM impairment could be explained by dopaminergic loss [54]. This statement is supported by recent neuroimaging data that show a significant positive correlation between ToM stories and Fluorodopa uptake in the right thalamus and the left putamen in PD patients [55]. Some previous studies in PD have suggested that ToM deficits may emerge at more advanced stages of the disease, specifically in those patients in whom the degenerative process has extended beyond the dopaminergic pathways, but not in early-stage patients where neuronal loss appears to be confined to the nigrostriatal and mesolimbic dopaminergic systems [56,57]. In any case, to date, there are no neuropathological studies confirming this hypothesis. 

On the other hand, cognitively normal PD patients did not show differences in SC performance in comparison with HC. Previous studies have shown that SC appears to be preserved in de novo cognitively normal PD patients [55], so it seems that SC is preserved as long as cognition is unimpaired. In this regard, it has been shown that deficits in cognitive functioning can predict future SC deficits [58], and SC severity is significantly associated with cognitive impairment [59]. 

As in previous research, cognitive ToM mainly correlated with language [60], attention [61], and executive functions [62,63]. Cognitive ToM and executive functions have been related to the associative circuit of the basal ganglia in PD involving the dorsal anterior cingulate cortex, the dorsomedial prefrontal cortex, and the dorsal striatum/caudate. Impairment of this circuit would lead to cognitive ToM dysfunction, whereas impairment of the affective ToM component would appear to be due to limbic network impairment, including the ventromedial prefrontal cortex and orbitofrontal cortex, the ventral anterior cingulate cortex, the amygdala, and the ventral striatum [16,64].

Even though we have shown that both cognitive and affective ToM are impaired in PD-MCI, the fact that PD-MCI patients only show impairment in affective ToM when comparing with HC and not with PD-CN, suggests initial cognitive ToM impairment followed by the addition of affective ToM worsening. 

In comparison to previous studies in non-demented PD [24,26], we did not find impairment in FER. This can be explained by the use of BFRT as a potential prerequisite to analyze FER [65,66], which may have functioned as a confounder in previous studies. Additionally, the exclusion of patients with severe clinical depression may explain this result. Depression has been described as a possible confounder when analyzing FER [67] and its severity increases FER deficits [68]. Likewise, our PD patient sample did not show self-monitoring difficulties. This aspect probably shares neural correlates with affective ToM and FER, since both are associated with insula and orbitofrontal cortex disconnection from structures of the salience network [69,70].

Finally, gender and educational level have been identified as having an impact in SC in the general population [71] and a weak effect on PD [60,72]. Although our sample has been matched for both variables, it is noteworthy that the inclusion in the analysis of these variables as potential confounders did not modify our results.

The main strength of this study lies in the administration of a comprehensive neuropsychological assessment and a broad SC evaluation to patients with homogeneous age and disease duration, the exclusion of severe depression, and the comparison with a healthy control sample. Nevertheless, our study limitation is that the small sample size could limit the generalizability of the results. In addition, we did not perform a sample size analysis, the number of subjects was chosen on the basis of previous studies.

## 5. Conclusions

Our findings indicate that SC is normal in cognitively unimpaired and mid-stage PD patients without clinically relevant depression, whereas a decline in affective and cognitive ToM seems to be linked to the presence of MCI. Overall, our results increase the evidence of SC as an important aspect of PD likely associated with the onset of cognitive impairment and emphasize the importance of evaluating these functions in this population.

## Figures and Tables

**Table 1:** Demographic, clinical, and neuropsychiatric variables.

	PD-CN M (SD) *n* = 33	PD-DCL M (SD) *n* = 19	HC M (SD) *n* = 27	*X*^2^, *F* or *t*	*p* Value
**Age, years ^c^**	68.0 (5.4)	67.4 (5.1)	66.4 (4.6)	2.65	0.27
**Females, *n* (%) ^e^**	12 (36.4)	3 (15.8)	11 (40.7)	3.45	0.17
**Education level, years ^c^**	14.7 (4.8)	12.2 (4.9)	14.7 (3.9)	4.48	0.11
**Disease duration, years ^d^**	5.2 (2.1)	6.4 (1.9)	n/a	3.92	0.053
**LEDD ^d^**	573 (316)	687 (184)	n/a	1.87	0.11
**MDS-UPDRS III ^d^**	17.9 (7.6)	24.6 (7.5)	n/a	8.73	0.005 ^a^
**GDS-30 ^c^**	7.2 (5.9)	8.9 (5.1)	5.8 (4.4)	3.63	0.16
**SAS ^c^**	11.0 (6.4)	16.1 (7.7)	6.3 (4.4)	12.9	0.002 ^a,b^

Note: ^a^ Significant differences between PD_MCI and PD_CN in post hoc test. ^b^ Significant differences between PD_MCI and HC in post hoc test. ^c^ ANOVA test used. ^d^ *t*-test used. ^e^ Chi-square used. PD-CN = Parkinson’s disease cognitively normal; PD-MCI = Parkinson’s disease with mild cognitive impairment; HC = healthy control; MDS-UPDRS: Movement Disorder Society-Sponsored Revision of the Unified Parkinson’s Disease Rating Scale; LEDD: Levodopa equivalent daily dose; GDS = Geriatric Depression Scale; SAS = Starkstein Apathy Scale.

**Table 2:** Social cognition assessment according to classification groups.

	PD-CN M (SD) *n* = 33	PD-MCI M (SD) *n* = 19	HC M (SD) *n* = 27	*X* ^2^	*p* Value
**RMET**	20.2 (4.7)	18.4 (3.2)	22.6 (4.6)	8.43	0.02 ^b^
**ToM Stories, total**	50.5 (9.1)	43.5 (10.8)	51.4 (6.1)	6.85	0.03 ^a,b^
**ToM Stories, sequencing**	29.5 (6.8)	25.4 (7.6)	30.0 (4.9)	5.11	0.07
**ToM Stories, questionnaire**	20.9 (2.8)	18.1 (3.9)	21.4 (1.7)	11.14	0.004 ^a,b^
**RSMS**	44.2 (12.6)	41.8 (6.9)	47.0 (8.4)	2.54	0.28
**KDEF (total score)**	46.7 (9.3)	44.1 (7.1)	49.6 (3.9)	2.23	0.32
**Neutral**	12.6 (4.3)	11.5 (3.7)	14.4 (2.4)	5.03	0.08
**Fear**	2.66 (1.8)	2.8 (1.1)	2.2 (1.5)	1.28	0.52
**Happiness**	7.8 (0.4)	7.7 (0.6)	7.8 (0.7)	4.30	0.11
**Disgust**	5.4 (2.2)	5.4 (1.5)	5.5 (1.7)	0.32	0.85
**Sadness**	4.7 (2.3)	3.9 (2.6)	5.6 (1.7)	2.02	0.36
**Surprise**	7.2 (1.5)	7.0 (1.3)	7.5 (0.5)	0.19	0.91
**Anger**	6.3 (1.3)	5.8 (2.2)	6.6 (1.3)	0.98	0.61

Note: Non-parametric variables. Kruskal–Wallis was applied. ^a^ Significant differences between PD-MCI and PD-CN in DSCF analysis. ^b^ Significant differences between PD-MCI and HC in DSCF analysis. PD-CN = Parkinson’s disease, Cognitively Normal; PD-MCI = Parkinson’s disease, mild cognitive impairment; HC = healthy control; RMET = Reading the Mind in The Eyes Test; ToM Stories = Theory of Mind Picture Stories Task; RSMS = Revised Self-Monitoring Scale; KDEF = Karolinska Directed Emotional Faces; DSCF = Dwass–Steel–Critchlow–Fligner pairwise comparisons post hoc test.

**Table 3:** Spearman rank correlations between neuropsychological evaluation and social cognition assessment.

		The Karolinska Directed Emotional Faces	Reading the Mind in the Eyes Test	Theory of Mind Picture Stories Task			Revised Self-Monitoring Scale
				Total	Sequencing	Questionnaire	
**Apathy**	SAS	−0.39	−0.47	−0.29	−0.30	−0.28	−0.32
**Attention**	Digit Span Forward and Backward	0.28 0.34	0.29 0.31	0.35 0.34	0.35 0.38	0.31 0.22	0.26 0.20
	TMTA	−0.30	−0.30	−0.52 ^a^	−0.48 ^a^	−0.54 ^a^	−0.15
**Executive function**	Stroop test inhibition	−0.50 ^a^	−0.30	−0.47	−0.47 ^a^	−0.41	0.11
	TMTB	−0.41	−0.25	−0.59 ^a^	−0.55 ^a^	−0.57 ^a^	−0.10
	Phonemic fluency	0.34	0.30	0.54 ^a^	0.51 ^a^	0.54 ^a^	0.01
**Language**	Boston Naming Test	0.44	0.34	0.63 ^a^	0.66 ^a^	0.48 ^a^	0.07
	Semantic fluency ^b^	0.24	0.33	0.39	0.34	0.42	0.01
**Memory**	CERAD	0.58 ^a^	0.52 ^a^	0.40	0.41	0.37	0.06
	WMS	0.47	0.39	0.56 ^a^	0.57 ^a^	0.48 ^a^	0.02
**Visuospatial function**	JLO	0.29	0.23	0.36	0.35	0.40	−0.03
	VOSP	0.17	0.25	0.33	0.35	0.33	−0.08
	ROCF	0.32	0.25	0.37	0.37	0.32	0.16

Note: ^a^ Statistically significant results after Bonferroni adjustment. ^b^ Parametric variable. Pearson correlation coefficient was used. TMTA = Trail Making Test A; TMTB = Trail Making Test B; CERAD = Consortium to Establish a Registry for Alzheimer’s Disease; WMS = Wechsler Memory Scale; JLO = Judgment of Line Orientation; VOSP = Visual Object and Space Perception Battery; ROCF = Rey–Osterrieth complex figure; SAS = Starkstein Apathy Scale.

## Data Availability

The data that support the findings of this study are available from the corresponding author, [C.G.S.], upon reasonable request. The data are not publicly available due to their containing information that could compromise the privacy of research participants.
